# Monthly Summary

**Published:** 1894-07

**Authors:** 


### Monthly Summary.

DEATH AFTER TAKING GAS.—At the City Coroner's Court. Mr. S. F. Langham held an inquest with reference to the death of Frank Lee, aged 25, manager to a colonial merchant, and latety living at Crowhurst Road, Brixton. Henry Creasy, dentist, 88 Newgate Street, City, deposed that on monday the deceased came to consult him with reference to having a tooth extracted with gas. He appeared to be in good health, and was put under gas by Dr. Adams, of Aldersgate street, who had previously examined him. He seemed to take the gas very well, and witness extracted the tooth. He went on breathing for about a third of a minute and then the breathing became stertorous. The doctor at once pulled the deceased's tongue forward, and he was afterward taken out of the chair and artificial respiration was resorted to. Ether was also injected, but finding that artificial respiration was of no avail, tracheotomy was performed. This also proved unsuccessful, as the throat was full of mucus, which seemed to impede breathing. After half an hour's work artificial respiration was given up, the deceased being found to be dead. Witness had carried on buisness in the city for the last twenty years, and had been in the habit of extracting teeth under gas during the whole of that time. Dr. Adams had always administered the gas, and with successsful results.

By the Coroner.—The deceased, in witness' opinion was an exceptionally good patient for gas. Dr. John Adams 180 Aldersgate street, stated that he had administered gas at Mr. Creasy's establishment during the last twenty years, and had to deal with about 40,000 cases. Of that number the present case was the first that had proved fatal. He saw nothing about the deceased to attract suspicion that he was not in a fit state to undergo the administration of gas. He seemed exceptionally strong. Dr. Norman Moore, lecturer on medicine at St. Bartholomew's Hospital, who had made the *post-mortem* examination, stated that all the organs were healthy, but he found no air in the lungs and they were engorged with blood. There was also much thick mucus in the bronchial tubes, and death was caused by asphyxia. In reply to the coroner, Dr. Moore said he believed that every thing had been done for the deceased that was possible. The jury returned a verdict of "Accidental suffocation," and added that no blame attached to the dentist or doctor.—*Dental Record.*

CARBORUNDUM.—A new compound, which seems destined to be of much practical importance, is now prepared on a manufacturing scale under the name of "carborundum." This substance resulted from the efforts of Mr. E. G. Acheson, to produce crystallized carbon for abrasive purposes. He has apparently produced an excellent abrasive material, though it is not composed entirely of carbon, as he expected it to be. The compound is made by heating together, in an electric furnace, sand, coke and salt. As obtained it consists of small crystals, so small, that the greater number of them pass through a seive having 2,590 meshes to the square inch The substance is very hard, very infusible and very difficult to burn. It is believed by its discoverer to be as hard as a diamond. It has been successfully used to polish diamond, glass, porcelain, hard iron and steel. It promises to be a very valuable substitute for emery and corundum in all such work. The substance is a chemical compound of carbon silicon.—*Items of Interest.*

---

THE FOURTH MOLAR.—"The fourth molar of complete form is sometimes found in the negro of the United States, and is well arranged in the arch. In the white races, however, it is nearly always a mere rodimentary peg, rarely or never being of typal form and well arranged in the jaw. A case is reported, however, in which there were four extra molars, one on each side of each jaw in a white woman (*Cosmos*, Vol. XIV., p. 227.) The reduction of the jaws in the higher races has contributed to its obliteration, and this contraction is now going on and is crowding out the third molar. The occasional reversion of the fourth molar is interesting, and cases where it presents in full form in the arch are of especial interest to the evolutionist as exhibiting a return to a condition that takes us back even of the higher apes which have the same dental formula as man."—*Dr. A. H. Thompson in Dental Headlight.*

HOW TO POLISH PORCELAIN TEETH.—It is frequently necessary to grind a porcelain tooth, to transform a central incisor into a lateral incisor, a cuspid into a bicuspid. More often one is obliged to grind a tooth to get it into the space it is to fill. I have tried several methods, but none have equaled, either in efficiency or in the rapidity with which it can be done, this process: First grind and shapen the tooth on the lathe, after which it is easy to remove all the marks of the lathe wheels and make the tooth very smooth by using an emery disk with the engine, following with a cuttle-fish disk. Then polish the tooth on the lathe with a felt wheel, using as a powder pulverized pumice stone, and you will be pleased with the splendid appearance of your tooth.

To remove the artificial glaze from a tooth, or crown, it is only necessary to use the paper disks on the engine and polish on the lathe as before mentioned.

If care is given, the work can be done in less than five minutes, and even if the tooth be of our American make, I doubt if an experienced eye would detect the grinding short of careful scrutiny.—J. VAN PELT WICKS, D. D. S.—*Items of Interest.*

---

SOLDERING ALUMINUM—By means of the alloys mentioned below, aluminum or other metals such as iron, tin plate, zinc, copper, brass, nickel, it is said, can be rapidly and easily soldered, either with the brazing iron or blow pipe. Aluminum can also be soldered to any of the above metals; the material is cheaper than any heretofore employed, gives a solid joint, and does not injure the metal by oxidation or otherwise: (1) Unalloyed pure tin, melting point 250°; (2) tin 1,000, lead 50, melting point 280° to 300°; (3) tin 1,000, zinc 50, melting point 280° to 320°; (4) tin 1,000, copper 10 to 15, melting point 350° to 450°; (5) tin 1,000, nickle, 10 to 15, melting point 350° to 450°; (6) tin 900, copper 100, bismuth 2 to 3; melting point 350° to 450°. The first three do not color aluminum, and can be used for ornamental and and artistic objects. Four and five are yellowish in color, but have the advantage of higher melting point and greater strength and hardness, and suggest the possibility of using aluminum for various articles and purposes for which hammered, coated or enameled iron, tin plate copper, zinc, lead, etc., are now used. The *Journal* of the Society of Chemical Industry says the last alloy can be made to assume any tint of yellow by varying the proportion of copper, and is therefore, suitable for soldering aluminum bronzes; the proportion of bismuth is adjusted so as to keep the melting point suitable for the use of the brazing iron.—*Items of Interest.*

---

ANÆSTHESIA.—At the Congress of the Surgeons of Germany, a table of statistics dealing with anæsthetics was presented for the fourth year. 51,846 cases are recorded: 32,745 with chloroform; 11,617 with ether, 3,896 with chloroform and ether; 760 with chloroform and ether and alcohol; 2,769 with bromide of ethyl. The average of fatal accidents is 1 in 2,587, and for chloroform 1 in 1,924. In the four years, 163,493 cases are recorded and 61 deaths. The average being with chloroform, 1 in 2,655. with chloroform and ether, 1 in 8,014; 1 in 4,890, chloroform, ether and alcohol; 1 in 26,268 with ether. Indeed with the last drug, but one death is recorded and it is pleasant to find that the number of cases in which ether is given has increased year by year. The fact that deaths have occurred even when Pictet's chloroform is used, tends to show that fatalities cannot in the absence of other causes, be assigned to the presence of impurities in the drug.—*London Dental Record.*

---

A DENTAL REVOLT.--In France, a dentist is a necessary legal appendage to every match factory, that the work-people may be protected against phosphor-necrosis. But as the dentist does not depend upon the good will of those who are his patients for his practice, he does not always exhibit the gentleness and politeness that is desirable. So in one establishment they protested against his brutality in an effectual manner. They mobbed him until he resigned.

OXIPHOSPHATE.—The antiseptic value of oxiphosphate is not sufficiently considered. Did you ever see caries progress under oxiphosphate? In cavities where the dentine is soft, crumbling or peeling up over the pulp in leathery layers, so that if you are not extremely careful you will expose the pulp, by removing the more advanced and disorganized portion of the walls, and then filling with oxiphosphate, you will find, in three months, all this covering of the pulp firm. Even though this covering had been so loose as to cause toothache, by first introducing, on cotton, chloroform tinctured with oil of cloves and carbolic acid, with a little tannin, this pain will almost immediately subside. In a few minutes, if you remove the caries as far ar you can without disturbing the pulp, you may safely fill with oxiphosphate. If the pulp is quite or nearly exposed, first place over it a little Canada balsam, on a sma[illegible] piece of paper. The cavity will be prepared for more thorough preparation and filling in three months. At this time, as much more of the carious portion must be removed as is consistent with the safety of the pulp; none of the dentine immediately over it should be disturbed, whether homogeneous or in layers; then refill with the cement plating this with alloy or gold, either before or after the cement has hardened.

The antiseptic, hardening, healing, bleaching and anti-thermal character of the oxiphosphate is so great that you will have better results in filling large cavities if most of them are made with it, though, of course the surface is more durable if covered with metal.—*Items of Interest.*

---

THE EARLIEST HOSPITAL ON RECORD.—In ancient times when disease overspred a nation, the churches did what they could for its alleviation and arrest. It was in obedience to the Sibyl's command that those physicians of Epidaurus arrived to assistthe inhabitants of plague-stricken Rome and to found on the Island of the Tiber the first general hospital of which there is any authentic record. It has long been in ruin. The nature of its position and the fact that it is nearly always under the Tiber's waves render it difficult to obtain an illustration.—*Lancet.*

CAPPING DENTAL PULPS.—I have a method of capping dental pulps, whicn has proved successful in my practice. I have saved 90 per cent of pulps by this method. About three years ago a lady called at my office to have a first lower molar extracted. Instead I inserted carbolic acid on a pellet of cotton, and let it remain about five minutes, while I prepared about three parts cement to one part of iodoform, and as much carbolic acid as it would retain. I removed the pellet of cotton, and immediately introduced the cement. In eight days I removed the filling, and to my surprise this immediately caused intense pain. I must confess I thought this a strange condition. I syringed the blood from the cavity, touched the pulp with carbolic acid and prepared the cavity for a permanent filling. Then filled with cement prepared as in the first instance, and filled the cavity, capping with amalgam. I saw her a short time since, and she assured me that the tooth was as good as any she possessed, and had never given any trouble since it was filled two years and a half ago. I have ever since employed this method of capping exposed pulps, and the result has been good.—*G. L. Mitchell, Bay St. Louis, Miss.*

---

"AMERICAN DENTISTS."—Prof. Park of Bufflo, had a dental experience in Cairo, Egypt, last winter. He saw the sign "American Dentist" on a door plate, and ventured in. He found that the proprietor was a swiss, born in Zurich, educated in Germany, and trained in France. He spoke German and French, but had no knowledge of English, and had never been in America. He based his claim to being an American dentist upon the fact that he was possessed of a set of operating instruments made in America. The European woods are full of such "American dentists."—*Dental Practitioner and Advertiser.*

